# Insights into the Functions of a Prophage Recombination Directionality Factor

**DOI:** 10.3390/v4112417

**Published:** 2012-10-24

**Authors:** Gaël Panis, Nathalie Franche, Vincent Méjean, Mireille Ansaldi

**Affiliations:** Laboratoire de Chimie Bactérienne CNRS UMR7283, Institut de Microbiologie de la Méditerranée, Aix-Marseille University, 31 chemin Joseph Aiguier, 13402 Marseille cedex 20, France; Email: Gael.Panis@unige.ch (G.P.); nfranche@imm.cnrs.fr (N.F.); mejean@imm.cnrs.fr (V.M.)

**Keywords:** prophage, lysogeny, recombination directionality factor, integrase, excisionase, response regulator, prophage induction, random mutagenesis, site-directed mutagenesis, protein-protein interaction

## Abstract

Recombination directionality factors (RDFs), or excisionases, are essential players of prophage excisive recombination. Despite the essentially catalytic role of the integrase in both integrative and excisive recombination, RDFs are required to direct the reaction towards excision and to prevent re-integration of the prophage genome when entering a lytic cycle. KplE1, HK620 and numerous (pro)phages that integrate at the same site in enterobacteria genomes (such as the *argW* tRNA gene) all share a highly conserved recombination module. This module comprises the *attL* and *attR* recombination sites and the RDF and integrase genes. The KplE1 RDF was named TorI after its initial identification as a negative regulator of the *tor* operon. However, it was characterized as an essential factor of excisive recombination. In this study, we designed an extensive random mutagenesis protocol of the *torI* gene and identified key residues involved in both functions of the TorI protein. We show that, in addition to TorI-TorR protein-protein interaction, TorI interacts in solution with the IntS integrase. Moreover, *in vitro*, TorR and IntS appear to compete for TorI binding. Finally, our mutagenesis results suggest that the C-terminal part of the TorI protein is dedicated to protein-protein interactions with both proteins TorR and IntS.

## 1. Introduction

The name bacteriophage encompasses all bacterial viruses, including temperate phages which have the particularity to integrate their genomes into their hosts, becoming prophages. The physiological state of the host determines the type of infection, either lytic or lysogenic. For example, in lambda, a starved host or a multiplicity of infection (MOI) higher than two, favors lysogeny [1,2]. The prophage is passively replicated as part of the host chromosome as long as conditions are not threatening to the host, in which case the prophage shifts to a lytic development [3,4,5]. Site specific recombination (SSR) constitutes a key step in lysogenic development since it is required for integration as well as for excision of the prophage genome [6]. This reaction is mediated in both directions by a specific recombinase, called integrase, that belongs either to the tyrosine or the serine recombinase families [7,8]. Whereas host factors can modulate the efficiency of the integrase mediated reactions, most of the time directionality is driven by recombination directionality factors (RDF) or excisionases [9,10,11,12]. In lambda, the RDF protein not only directs the reaction towards excision but also prevents reintegration of the excised phage genome [13,14,15]. 

Bacterial genomes are parasitized by prophages and prophage remnants, which can constitute up to 20% of the host genome. A pan-genomic study of 20 *Escherichia coli* genomes revealed that unique prophage genes are more abundant than core genes present in all 20 genomes [16]. Prophages are thus an important vector of bacterial genome evolution. While integrated into the host genome prophages also undergo rapid evolution, occurring mostly through homologous recombination and frequently leading to the loss of lytic genes [17,18]. The resulting prophages are not infectious anymore, however, they may conserve features that suit the host. In *Escherichia coli *K12, 10 prophage regions have been identified [19]. We studied one of them in particular, the KplE1 (or CPS53) prophage. This latter is inserted into the *argW* tRNA gene at 2,474 kb on the *E. coli* chromosome and contains 16 ORFs. Most have unknown functions, whereas we previously characterized the role of the first gene *intS* and the last one *torI* in site-specific recombination [20,21]. Interestingly, various (pro)phages that insert at the same tRNA gene locus share a highly conserved recombination module that comprises the *attL* and *attR* sites and the IntS integrase and the TorI RDF genes [21]. The identity even reaches 100% with the RDF proteins of HK620 and Sf6, named HkaC and P18, respectively [22,23].

The TorI protein (for Tor Inhibition) was originally identified using a genetic screen as a negative regulator of the *torCAD* operon that encodes the trimethylamine oxide reductase respiratory system in *E. coli *[24]. Despite its role as an inhibitor of the TorR response regulator, TorI was then genetically and structurally characterized as the RDF of the KplE1 prophage [20]. Recently, we also identified the host-encoded stress-responsive molecular chaperone DnaJ as an active participant in KplE1 prophage excision. DnaJ is recruited by TorI and stabilizes its tridimensional structure which has for consequence to increase TorI affinity for its specific binding sites on *attL* [25,26].

In this study, we designed an extensive random mutagenesis protocol of the *torI* gene to identify critical residues involved in the anti-response regulator (anti-RR) and/or the excisive recombination activity functions of the TorI protein.

## 2. Results and Discussion

### 2.1. Tester Strain and Random Mutagenesis of the torI Gene

**Figure 1:**
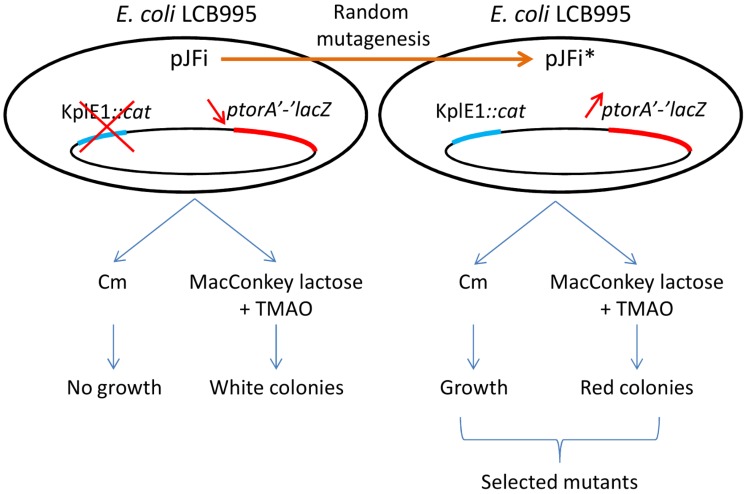
Strategy to study TorI functions.

In order to identify critical residues involved in the anti-RR and/or the excisive recombination activity of the TorI RDF, we designed a tester strain that can report both activities. Strain LCB995 contains a *torA'-'lacZ* fusion as well as a *cat* cassette inserted into a non-coding region of the KplE1 prophage [20,27]. When this reporter strain was plated onto MacConkey lactose plates in the presence of 10 mM TMAO to induce the *tor* operon promoter, the colonies formed turned red, as the β‑galactosidase was produced [27]. In contrast, when this strain was transformed with a multicopy plasmid encoding a wild-type version of the *torI* gene (pJFi plasmid), which allows inducible TorI production in the presence of 1 mM IPTG, the resulting colonies remained white on the same medium due to the anti-RR activity of TorI. On the other hand, the RDF activity of TorI was monitored with the same tester strain containing pJFi plated onto chloramphenicol (Cm) containing plates in the presence of 1 mM IPTG. As described before [20], expression of the *torI* gene is sufficient to promote KplE1 excision, and thus renders the cells chloramphenicol sensitive. Since colonies on agar plate arose from single cells, colonies are not forming from cells that have excised the KplE1 prophage. We thus have set up an experimental procedure to screen a mutant library of TorI that may be affected in two different activities (Figure 1). 

The mutant library of *torI* alleles was generated by extensive random PCR-mutagenesis of the *torI* gene (201 base pairs) carried by plasmid pJFi (see the Experimental Section). About 3,000 colonies were screened on reporter (MacConkey plates containing lactose and TMAO) or selective media (LB plates containing chloramphenicol), and colonies that presented a red color on MacConkey lactose plates containing TMAO or that could grow in the presence of chloramphenicol, were selected. A final set of 42 clones reproducibly altered in one or both TorI activities was selected for further investigation (Figure 1). To make sure that the observed phenotypes were not due to chromosomal mutations, all plasmids were extracted and used to transform the tester strain. Direct Sanger sequencing of the plasmids carrying the mutated alleles allowed the identification of the mutations. In total we isolated 18 distinct mutations, of which 16 were due to a point mutation leading to a single amino-acid substitution (Table 1). Overall, the mutagenesis showed no obvious mutational bias with mutations dispersed all along the primary sequence and a wide diversity in the mutations that we obtained with 15 transitions, four transversions, one deletion and two extensions due to a frameshift on the stop codon. No redundant mutations were observed, in total 20 different positions out of 66 were affected, most of them were changes in residues located on the surface of the protein (with the exception of Ile_16_, Trp_47_ and Phe_56_), and only one substitution has impacted the overall structure of the protein (L5P, Table 1 and text below). Thereafter, pJFi* designates a plasmid carrying a mutated allele, and TorI* indicates a mutant protein.

A preliminary *in vivo* quantification of TorI activities was performed (Table 1). The anti-RR activity of the TorI mutants was estimated by monitoring β-galactosidase activities of strain LCB995 containing pJFi*. This activity reports the expression of the *torA'-'lacZ* fusion in the presence of TMAO. On the other hand, the RDF activity of TorI mutants was measured in the same strain by counting colonies able to grow on chloramphenicol plates relative to colonies counted on ampicillin plates. Results are expressed as the ratio of ampicillin-resistant/chloramphenicol-resistant colonies, and reflect the ability of the TorI mutants to promotes KplE1 excision. We identified two classes of mutants: one class contained mutants affected in the anti-RR activity only, whereas the second class was composed of mutants affected in both activities. As a result, we did not isolate mutants affected in the RDF activity only. In recent studies [20,26], we also designed several mutants by site-directed mutagenesis which were included in Table 1 (^d^ mark) and were further analyzed together with the mutants generated by random mutagenesis.

**Table 1:** Substitutions in TorI mutants.

Plasmid ^a^	Mutation	Substitution	RDF activity ^b^	Anti-TorR activity ^c^
pJFi	N/A	N/A	+ + + +	+
pJFi-L5P	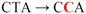	Leu5 → Pro	+ + + +	−
pJFi-S9L	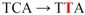	Ser9 → Leu	−	−
pJFi-D12Y-D35G	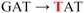	Asp12 → Tyr	−	−
	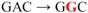	Asp35 → Gly		
pJFi-F15L	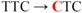	Phe15 → Leu	−	−
pJFi-I16V	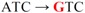	Ile16 → Val	+ +	−
pJFi-M17V	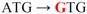	Met17 → Val	+ +	−
pJFi-F22I	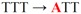	Phe22 → Ile	−	−
^d^ pJFi-Y28F	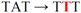	Tyr28 → Phe	−	−
^d^ pJFi-Y28S	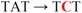	Tyr28 → Ser	−	−
pJFi-P37L	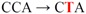	Pro37 → Leu	+	−
pJFi-H43Y-C54R	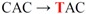	His43 → Tyr	+	−
	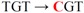	Cys54 → Arg		
pJFi-R45STOP	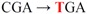	Arg45 → STOP	−	−
^d^ pJFi-R45Q	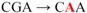	Arg45 → Gln	+	−
^d^ pJFi-R45K	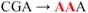	Arg45 → Lys	+ +	−
pJFi-A46V	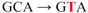	Ala46 → Val	−	−
pJFi-A46T	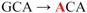	Ala46 → Thr	−	−
pJFi-W48R	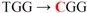	Trp48 → Arg	−	−
pJFi-E55G	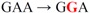	Gln55 → Gly	+ + + +	−
pJFi-F56L	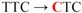	Phe56 → Leu	+ + + +	−
^d^ pETsI-L61S	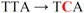	Leu61 → Ser	ND	ND
^d^ pETsI-R63C-A64S	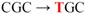	Arg63 → Cys	ND	ND
	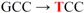	Ala64 → Ser		
pJFi-N65Y	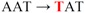	Asn65 → Tyr	+ + + +	−
pJFi+18	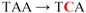	+18 residues ^e^	+	−
pJFi+24	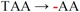	+24 residues ^f^	+ + + +	−

^a^ All *torI* alleles of the pJFi* plasmid series have been sub-cloned into the pET-22(+) vector leading to the pETsi* series; ^b^ RDF activities were estimated by the ratio of colonies Ap^R^/Cm^R^ and indicated as follows: ++++ 80 to 100% of the activity measured in the presence of the WT *torI* allele , ++ 1 to 10%, + 0,1 to 1% , and − <0,1% (corresponds to the activity observed in the presence of the empty vector pJF119EH); ^c^ Anti-RR activities were estimated by β-galactosidase activity measurements of cells grown in the presence of 10 mM TMAO and 1 mM IPTG. + indicates the activity of the WT *torI* allele, − refers to a null anti-RR activity; ^d^ Mutants obtained by site-directed mutagenesis [20,26]; ^e^ Additional residues: SGSSRVDLQACKLGCFGG; ^f^ Additional residues: KDPLESTCRHASLAVLADERRFSA.

### 2.2. Mapping of the Mutations that Affect TorI Activities

**Figure 2:**
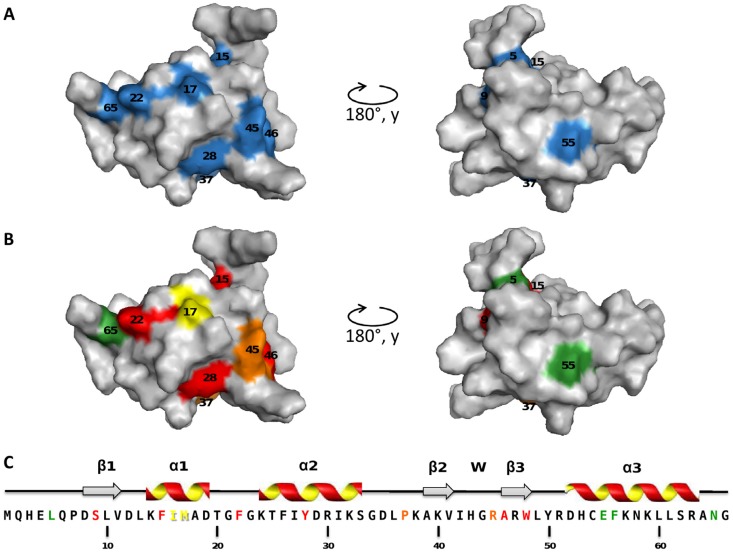
Mapping of the mutations that affect the anti-RR function (**A**) or the recombination directionality factors (RDF) activity (**B**) of TorI; Secondary structures of the TorI protein (**C**). Color code is as follows: *blue*, no anti-RR activity; *green*, 80 to 100% of the WT activity; *yellow*, 1 to 10%, *orange*, 0.1 to 1%, and *red*, no RDF activity.

To our surprise, all the mutations we obtained impaired the anti-RR activity of TorI, and these mutations map all over the surface of the protein (Figure 2A). In a previous work, we showed that TorI inhibited the transcriptional activator TorR through a direct protein-protein interaction without preventing TorR binding to its specific DNA targets, and we hypothesized that TorI was binding to a region of TorR that is important for RNA polymerase recruitment [24]. All the surface mutations that we obtained altered the anti-RR activity, suggesting that more than one face of the protein is involved in this function and that one face of the protein could bind to TorR and another to RNA polymerase. A vast majority of the mutants isolated affect surface residues, which are probably involved in protein‑protein interactions. One may have expected a more subtle effect on the anti-RR activity, however, our screen failed to select those. 

In contrast to the anti-RR activity, only a subset of mutations altered the RDF activity of TorI and most of these mutations map on the surface of the protein although the mutations are scattered along the primary sequence (Figure 2B). By this approach, we identified two regions of the protein involved in the excisionase function: one centered on helix 1 and another one comprising the helix 2 and the wing motif. The wing-helix is an atypical helix-turn-helix motif involved in DNA-binding commonly found in transcription factors and particularly in the OmpR family of response regulators [28,29]. Based on the work done on the lambda Xis protein, two separate functions are [22] required for the excisionase activity: (i) Xis interacts with DNA to position itself on the *X* sites on the *attR* recombination region, (ii) Xis interacts with Int to direct a proper positioning of the integrase for the excisive reaction [15]. In a previous work, residues involved in DNA binding were identified by NMR titration with a short DNA sequence and mapped onto the wing-helix motif [20]. Therefore, two positions of the TorI wing-helix motif were mutated (Tyr_28_ and Arg_45_) and the resulting proteins proved to be impaired in KplE1 excision, although Tyr_28_ mutants were more affected than Arg_45_ ones, suggesting a role for this region in DNA binding activity.

### 2.3. Characterization of the TorI Mutants

#### 2.3.1. Production and Stability of the Mutants for *in Vitro* Studies

As a preliminary quality check, we selected 14 single substitution mutants (only one substitution at position 28 and 45 were selected) and analyzed their respective production and stability by western‑blot. All the mutants were produced from the pJFi* plasmids in the presence of 1 mM IPTG. Crude extracts were run on a 16% SDS Tricine-PAGE and TorI* production was revealed using an anti-TorI polyclonal serum (see Experimental Section). Accordingly, all mutant proteins were produced at similar levels and the antibodies failed to reveal bands below the full size proteins indicating that all mutants have a similar stability *in vivo* as the wild-type protein (data not shown).

#### 2.3.2. *In Vitro* RDF Activity of TorI Mutants

**Figure 3:**
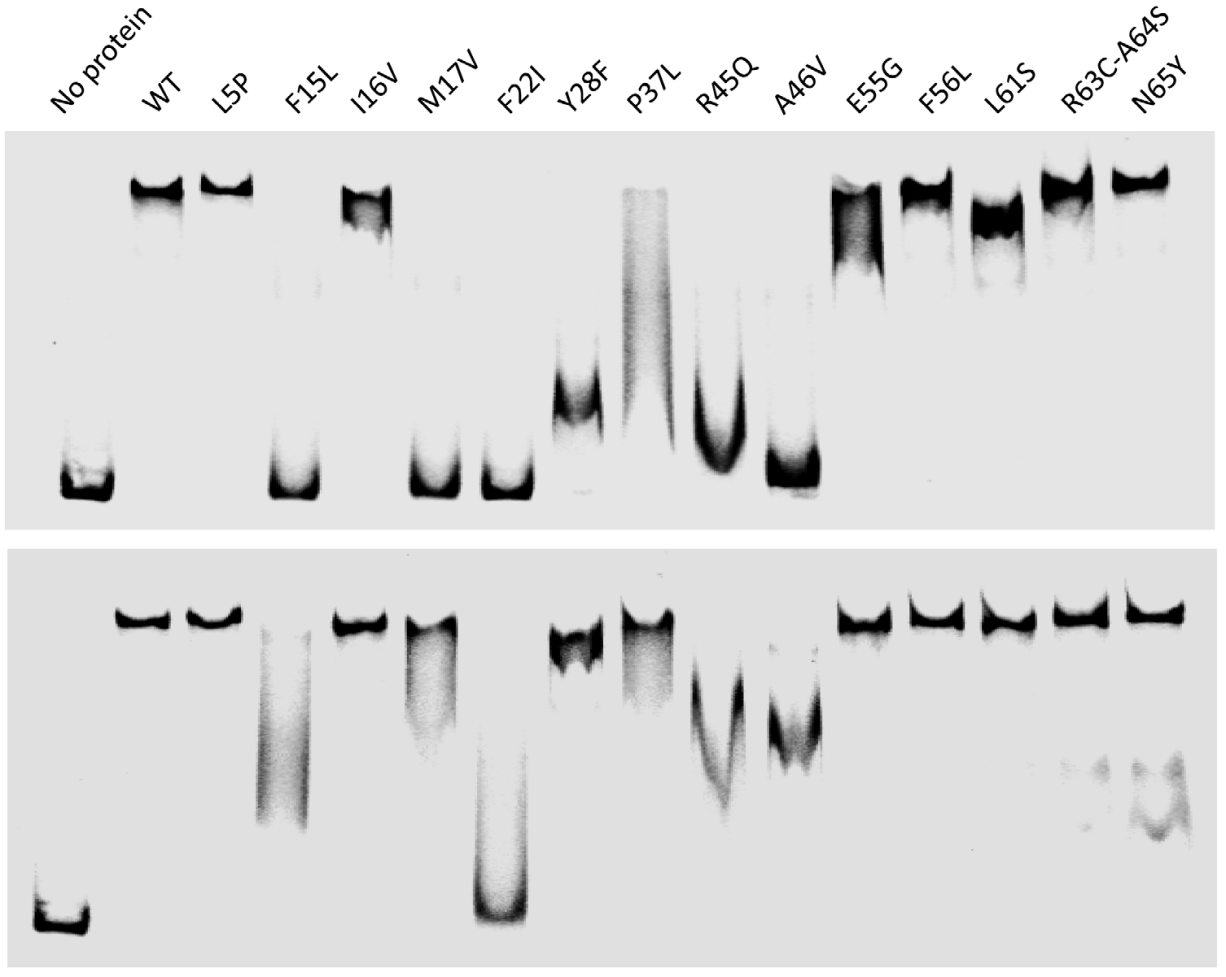
DNA binding activity of the TorI mutants. Electrophoretic mobility shift assay (EMSA) experiments were performed using the KplE1 *attL* recombination region that contains five cooperative binding sites for TorI [30]. Proteins were all used at a concentration of 10 µM whereas DNA concentration was either 20 nM (top) or 10 nM (bottom). When two *torI* alleles were available, only one was chosen for *in vitro* characterization, namely TorI-Y28F, TorI-R45Q, and TorI-A46V.

Most mutants isolated in this study, as well as those designed elsewhere [20,26], were produced from pETsI* plasmids, purified near homogeneity and analyzed *in vitro*. Only a few of them proved to be unstable upon purification (TorI-S9L and TorI-W48R). Since the RDF activity of TorI relies on its DNA binding activity, we first checked the ability of the mutants to bind to *attL*. Electrophoretic mobility shift assay (EMSA) were performed essentially as described [30], except that two different concentrations of DNA were used in this assay (Figure 3). 

At high DNA concentration (top panel), several mutants displayed reduced DNA binding activity. The mutations mapped either in the helix-turn-helix motif (F15, M17, F22, Y28), or in the wing region (R45, A46). Interestingly when the protein:DNA ratio was increased (Figure 3, bottom panel), most of these mutants were able to shift *attL *to a certain extent, with the exception of F22I. As TorI binding to the 5 TorI sites in *attL* is highly cooperative [30], smeary patterns were observed in some cases rather than intermediate shifts. This result indicates that point mutations in the DNA binding region of TorI affect binding to *attL* by reducing the affinity of the protein for its DNA substrate, and this effect can be often overcome by increasing the protein:DNA ratio.

#### 2.3.3. *In Vitro* Excisive Recombination

**Figure 4:**
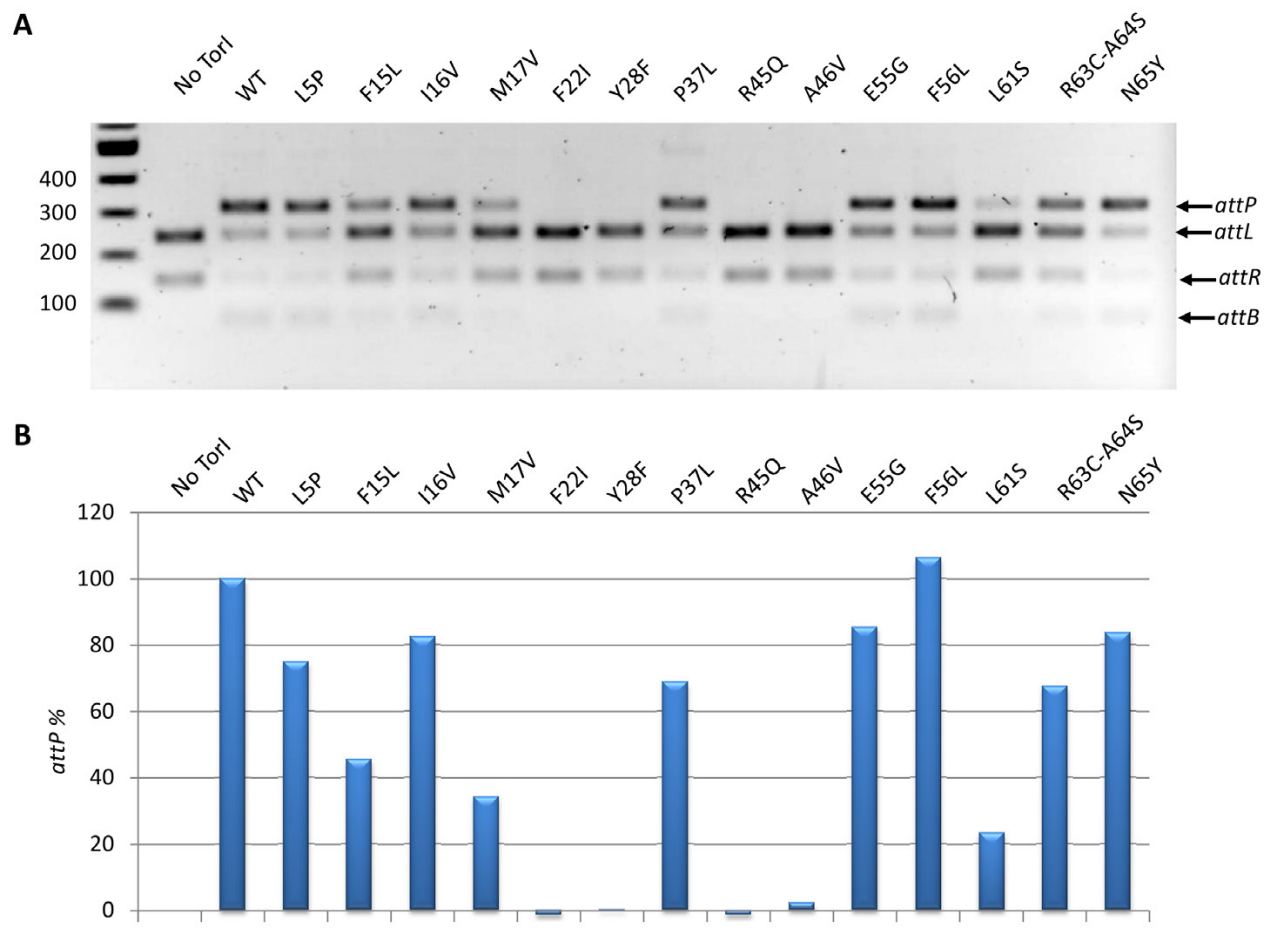
*In vitro* excisive recombination assay. Reactions were conducted as indicated in the Experimental Section with the same protein variants as in Figure 3. A, the agarose gel electrophoresis shows the relative migration of the substrates (*attL* and *attR*) and the products (*attP* and *attB*). B, the gel shown in A was scanned and the *attP* product quantified. Activities of the mutants are expressed as a percentage of the WT protein activity.

We further investigated the effect of the TorI mutations on the ability of the protein to promote excisive recombination *in vitro*. Under the *in vitro* conditions we used (see the Experimental Section), all mutants generally behaved the same way as *in vivo*, also some discrepancies could be observed probably due to the highest sensitivity of the *in vitro* assay (Figure 4). Four mutants (TorI-F22I, TorI‑F28F, TorI-F45Q, and TorI-A46V) were totally inactive *in vitro*, and the corresponding mutations all lie in the winged-helix structural motif (Figure 2). Interestingly, these same mutants were also largely impaired in binding to the *attL* substrate (Figure 3). In addition, three mutants (TorI-F15L, TorI-M17V, and TorI-L61S) showed significantly reduced *in vitro *activities. Among these, TorI-F15L and TorI-M17V were clearly affected in DNA binding to *attL* as well and the corresponding mutations lie in the first α-helix of the helix-turn-helix motif. In contrast, TorI-L61S exhibited an almost wild‑type DNA binding activity and the substitution occurred in the last α-helix of the protein, therefore excluding this region from the DNA binding domain. Together, these results are consistent with DNA binding to *attL* being the primary activity required for optimal RDF activity.

#### 2.3.4. Competition for TorR and IntS Binding on TorI Protein

**Figure 5:**
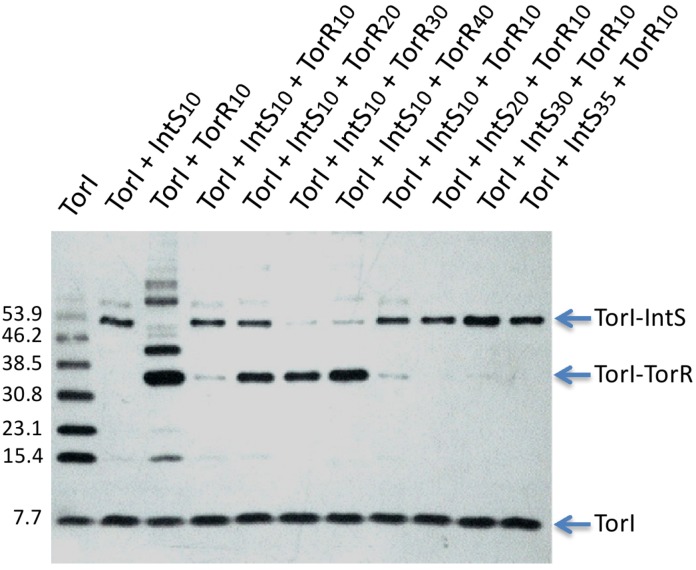
IntS and TorR bind to TorI and replace each other. A binding assay of TorI (10 µM) in the presence of various IntS and/or TorR concentrations, as indicated, was performed using BMH as a crosslinker (see the Experimental Section). Major TorI-IntS and TorI-TorR complexes are pointed by arrows.

Regarding the two functions in which TorI was shown to be involved, we then asked if the proteins involved in these functions, namely TorR and IntS could compete for the same TorI target. We have previously shown that TorI was able to bind to the C-terminal domain of the TorR response regulator [24], however an interaction with IntS, although suspected, was not yet identified. Indeed, when incubated in the presence of IntS, TorI was revealed in a band in which migration (~50 kDa) was compatible with a heterodimer TorI-IntS (IntS, 42.5 kDa and TorI, 7.7 kDa) (Figure 5, lane TorI+IntS10). Moreover, the presence of the IntS protein in this extra band of ~50 kDa was confirmed by mass spectrometry analysis and by using α-IntS antiserum (data not shown). According to what has been shown earlier [24], in the presence of TorR a major band corresponding to a 1:1 TorI:TorR ratio (~34 kDa) was detected with α-TorI antiserum. Additional bands may correspond to complexes with different TorI:TorR ratios (2:1, ~42 kDa; 4:1, ~57 kDa), and suggest that TorR can bind multiple forms of TorI. Remarkably, when IntS and TorR were incubated together with TorI and at equimolar concentrations (10 µM), only a faint band corresponding to the major complex TorI:TorR was detected, indicating that IntS has probably more affinity for TorI than TorR does. However, in the presence of molar excess of TorR (20, 30 or 40 µM), TorR was able to displace IntS, although not totally. On the other hand, when IntS was added in excess in the reaction compared to TorR, no more TorI:TorR complex was observed. Together, these results suggest that IntS and TorR bind to a similar region on TorI and that the complex formed between TorI and IntS is more stable than with TorR.

#### 2.3.5. TorI Mutants Binding to TorR and IntS

**Figure 6:**
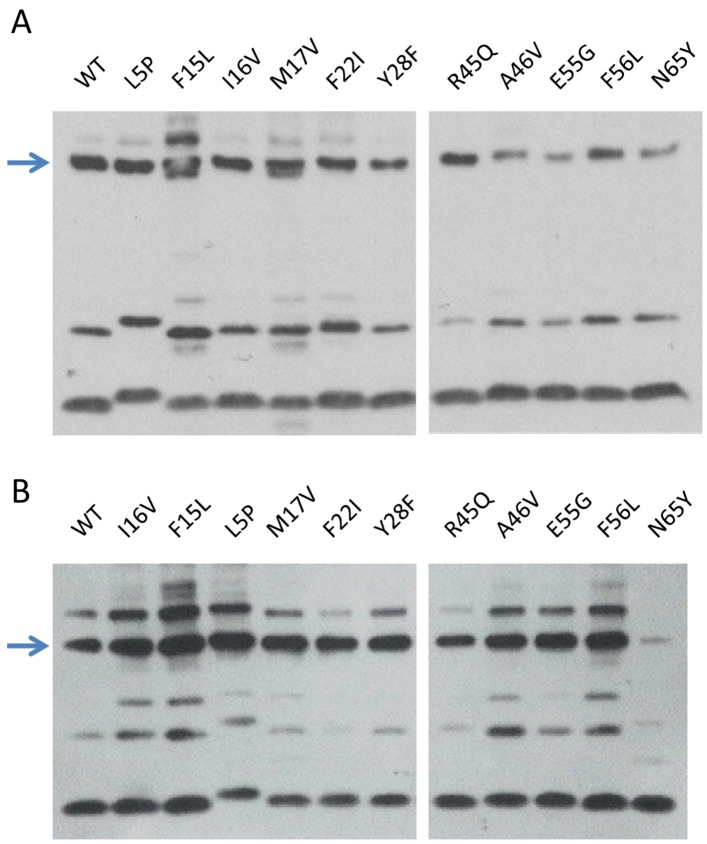
IntS and TorR binding to TorI mutants. A, binding assay of TorI mutants (10 µM) in the presence of IntS (10 µM). B, binding assay of TorI mutants (10 µM) in the presence of TorR (10 µM). Arrows indicate the major complexes.

As suggested by the competition experiments, TorR and IntS may bind a similar region on TorI. Our TorI mutant collection was thus assayed for binding with the two proteins (Figure 6). When assayed in the presence of IntS (Figure 6A), all TorI mutants were able to crosslink with IntS although, some, such as TorI-A46V, TorI-E55G and TorI-N65Y exhibited reduced binding. In contrast, binding with TorR (Figure 6B) indicated that TorI-N65Y, which has the mutation in the last amino-acid of the protein, did not bind anymore TorR. Together, these results suggest that the C-terminal part of the TorI protein is involved in both IntS and TorR binding.

## 3. Experimental Section

### 3.1. Strains and Media Used in This Study

Strains and plasmids used in this study are listed in Table 2. Strain LCB995 was constructed by transduction of the *cat* marker inserted in the KplE1 prophage between *yfdO* and *yfdP* (strain LCB970 [20]) into strain LCB620 carrying a *torA'-'lacZ* fusion [27]. Strains were grown in LB medium supplemented when necessary with ampicillin (50 µg.mL^−1^), chloramphenicol (25 µg.mL^−1^), TMAO (10% v/v) or IPTG (1 mM).

**Table 2:** Strains and plasmids.

Strains and plasmids	Characteristics	Sources
**Strains**		
MC4100	*araD139* (∆*lacIPOZYA-argF*) *U169 rpsL thi*	Casadaban
LCB620	MC4100 *torA8*::MudII 1734 (*torA'-'lacZ*, Km^R^)	[27]
LCB970	MC4100 *yfdO-cat-yfdP*	[24]
LCB995	MC4100 *torA8*::MudII 1734, *yfdO-cat-yfdP*	This work
LCB984	MC4100 *yfdO-kan-yfdP*	This work
BL21(DE3)	*E. coli* B F-* [lon] dcm ompT hsdS *(r_B_^−^m_B_^+^) gal λ(DE3)	Novagen
C41(DE3)	Derived from BL21(DE3)	[31]
**Plasmids**		
pBAD33	pACYC184 (ori p15A) vector containing a P_BAD_ promoter (Cm^R^)	[32]
pBtorR	*torR* coding sequence cloned into pBAD33	[33]
pJF119EH	pBR322 (ori *col*E1) containing the IPTG inducible promoter p*tac* (Ap^R^)	[34]
pJFi	*torI* coding sequence cloned into pJF119EH BamHI and EcoRI sites	[24]
pJFi* series	pJFi derived plasmids carrying *torI* mutated alleles	This work [20,26]
pET-22(+)	Promoter T7 containing vector (Ap^R^)	Novagen
pETsi	*torI* coding sequence with a Stop codon cloned into pET-22(+) NdeI and XhoI sites	[24]
pETsi* series	pETsi derived plasmids carrying *torI* mutated alleles	This work [20,26]

NB, all plasmids derived from pJFi and pETsi plasmids and carrying mutated *torI* alleles are mentioned in Table 1. Otherwise indicated, pJFi and pETsi derivatives were isolated or constructed in this study.

### 3.2. Random Mutagenesis

Error prone PCR was performed to generate mutated alleles of the *torI* gene. PCR was conducted with the primer pair torI_MunI (5'-TAC AAT TGC GGA GAT AGC ACT CAT GCA ACA C)/torI_BamHI (5'-TTG GAT CCT TAC CCA TTG GCG CGG CTT AAG AG), plasmid pJFi as a template and a classical Taq polymerase (GoTaq Promega, error rate 10^−6^). Three rounds of PCR were performed using as a template a 10^6^ dilution of the product generated by the previous round of PCR. After three rounds of PCR, products were purified, hydrolyzed with MunI and BamHI enzymes and ligated into the pJF119EH vector cut with EcoRI and BamHI. The plasmid library was then transformed into the tester strain LCB995.

### 3.3. Protein Production and Purification

IntS, TorI and IHF proteins were overproduced and purified to homogeneity as previously described [24,30]. TorI mutants were purified as the wild-type protein. All proteins were dialyzed in Tris-HCl buffer (40 mM, pH 7.6) containing 50 mM KCl and 10% glycerol. The protein concentrations were measured by densitometry with the wild-type TorI protein as a reference.

### 3.4. In Vivo Excision Assay

Strain LCB970 carrying *torI *encoding plasmids pJFi (7) was grown in LB medium until the OD_600_ reached 0.5 units (0.5 × 10^9^ cells.mL^−1^), and IPTG (1 mM) was added for 2 h at 37 °C under agitation. Culture dilutions were prepared and plated onto rich medium containing either 50 µg.mL^−1^ ampicillin or 25 µg.mL^−1^ chloramphenicol. Numeration of the colonies plated on both antibiotics was performed and the ratio of ampicillin-resistant/chloramphenicol-resistant colonies was calculated. Values represent the average of at least three independent determinations.

### 3.5. β-Galactosidase Assay

β-Galactosidase activities were measured on whole cells according to the method of Miller (1972); values represent the average of at least three determinations with a variation of no more than 10% from the mean.

### 3.6. *In Vitro* Excisive Recombination

Linear *att* sites were amplified by PCR with primer pairs attL-SpeI (5'-GAC TAG TTT CAA TCT GCT TAA CGG TGA GCA T)/attL-KpnI (5'-GGG GTA CCG CTA ATT GCA GGT TCG ATT CC) for *attL* (220 bp) and attR-XbaI (5'-GCT CTA GAG GTT TTA GGG ATA AAC ACA CAA GGA TG)/attR-IHF2 (5'-CTC TTA AGC CGC GCC AAT GG) for *attR* (135 bp), and then purified using Qiaquick PCR purification kit protocol (Qiagen). Reaction mixtures (25 µL) included linear *att* DNA sites (28 nM) in buffer containing 33 mM Tris-HCl pH 7.6, 33 mM KCl, 9 mM spermidine, 4 mM EDTA, 0.9 mg/mL^−1^ acetylated BSA and 7 % glycerol. IHF (0.3 µM), IntS (0.6 µM), and TorI (2.1 µM) were added as indicated in the figures legends. The reactions were carried out in optimized conditions at 30 °C for 2 h at an IHF:IntS:TorI protein ratio of 1:3:7. Reaction products were purified (Qiaquick kit, Qiagen) and analyzed on a 2% agarose gel electrophoresis. The gel was then scanned and the data analyzed using AlphaView software (Protein simple).

### 3.7. Electrophoretic Mobility Shift Assays (EMSA)

EMSA were carried out using purified proteins and fluorescently Cy5-labelled *attL* DNA fragment that was amplified by PCR using MG1655 chromosomal DNA as a template with the primer pair attL‑pro (5'-AAT GGA TAT AAC GAG CCC CTC C)/attL-ter-Cy5 (5'-CAT CGA GAA GGC GGT ATG GTT TTT C). DNA and purified proteins were mixed together at different concentrations (as indicated in figure legends) in the presence of 4 mg.mL^−1^ BSA and 0.5 mg.mL^−1^ calf thymus DNA (CT-DNA) in binding buffer (40 mM Tris pH 7.6, 85 mM KCl, 19% glycerol). Reactions were incubated for 30 min at 30 °C. DNA-protein complexes were then separated using a 6% non‑denaturing polyacrylamide gel (37:1 acrylamide:bisacrylamide ratio). A pre-migration step (1 hour at 160 V) was carried out to reduce ionic charges which may have destabilized the DNA‑protein complex. Samples were then loaded and left to migrate at 80 V during 30 min and then at 160 V for 2 more hours in 0.5× TBE (50 mM Tris, 45 mM Boric acid, 0.5 mM EDTA) running buffer. The gel was scanned using a FLA5100 (Fuji) scanner, using excitation wavelength of 635 nm (800 V scanning intensity) and emission wavelength of 665 nm. Data was analyzed using Multi Gauge (Version 2.3) software [35].

### 3.8. Cross-Linking Analysis

TorI protein was pre-incubated 10 min at 25 °C in the presence or absence of IntS and/or TorR proteins. Then, homobifunctional sulhydryl reactive agent bis(maleimido)hexane (BMH, 1mM, Pierce) was added and the reaction continued for another 30 min. Samples were ran on a 7%–16% Tricine‑SDS PAGE, transferred onto a nitrocellulose membrane and revealed by immunodetection with TorI antiserum.

## 4. Conclusions

In this work we describe an extensive mutational analysis of a small protein involved in prophage excision. Together, our results suggest that the C-terminal part of the TorI protein is somehow dedicated to protein-protein interactions, since this region seems to bind at least three different molecular partners, the TorR response regulator, the IntS integrase and, as shown before, the DnaJ cochaperone [25,26]. This is particularly relevant to the tridimensional structure of the TorI family of RDF proteins that contain a long and well defined α-helix at the C-terminus of the protein, which is not found in other RDF proteins in solutions [20].
